# Blood Transfusion in the 21st Century

**DOI:** 10.15190/d.2014.3

**Published:** 2014-03-31

**Authors:** Mark T. Friedman, Vaidehi Avadhani, Sandra Gilmore, Emilio Madrigal

**Affiliations:** Department of Pathology, Blood Bank and Transfusion Services; Blood Management, Bloodless Medicine and Surgery, Mount Sinai Health System, St. Luke’s, Roosevelt, and Beth Israel Hospitals, Icahn School of Medicine at Mount Sinai, New York, NY 10019, USA

**Keywords:** Blood substitutes, factor concentrates, hemovigilance, patient blood management, transfusion medicine education, transfusion safety

## Abstract

Blood transfusion is a common procedure in the hospital setting, and the safety of the blood supply has been vastly improved over the past few decades largely due to improvements in screening for viral transmissible diseases, especially human immunodeficiency virus (HIV) and viral hepatitis. However, more recent efforts to improve blood safety have focused on non-transmissible disease risks such as transfusion-related acute lung injury (TRALI), non-viral transmissible diseases such as bacterial contamination of blood products (especially platelet components which are stored at room temperature) and Chagas disease (a parasitic disease caused by Trypanosoma cruzi), and prion transmissible agents (e.g., variant Creutzfeldt-Jakob disease, also known as the agent of mad cow disease) as well as more recently-recognized transmissible viral disease risks such as West Nile virus. Appropriate blood utilization has also come under more intense scrutiny in recent times due to healthcare costs and the recognition that many blood transfusions are given under circumstances in which the benefit to the patients is unclear and may be potentially harmful due to the above risks as well as the emerging concept that blood transfusions may cause long-term damage to the immune system resulting in worse patient morbidity and mortality outcomes. Toward that end, accreditation agencies such as the Joint Commission and the American Association of Blood Banks (AABB) are advocating for healthcare organizations to implement appropriate patient blood management strategies. This review will examine these issues along with newer blood safety technological innovations and further highlight contributing studies from our institutions.

## SUMMARY

1. Introduction
2. Safety of the blood supply
3. Quality of the blood supply
4. Transfusion medicine education
5. Patient blood management
6. Future technology
7. Concluding remarks

## 1. Introduction

Blood transfusion is one of the most common procedures performed in hospitals. Accordingly, it was reported that there were nearly 15 million whole blood and red blood cell units transfused in the United States alone in 2008, representing a nearly 6% increase in transfusions from the prior surveyed year of 2006^1^. This is because anemia is a common comorbidity that can be found in a variety of patients, including patients with cancer, patients undergoing surgical procedures or hemodialysis, patients with autoimmune or bone marrow disorders (such as myelodysplastic syndrome), patients with congenital anemia (such as sickle cell anemia and thalassemia), trauma patients, obstetrical patients, and patients with nutritional deficiencies, such as iron deficiency. While it is certainly known that anemia can have adverse effects on patients, particularly in cardiovascular and neurocritical care patients^2-4^, what is becoming more evident is that correction of anemia via blood transfusion is not beneficial to many patients, and in fact, may be more harmful, unless the patient is acutely symptomatic (e.g., shortness of breath, low blood pressure, rapid heart rate, dizziness, or chest pain) from the anemia. Thus, it is considered problematic that many blood transfusions are administered unnecessarily, driving up healthcare costs, expending a valuable resource (i.e., blood components) in short supply (because only a minority of eligible blood donors in the U.S. give blood), and exposing patients to all the risks and complications (like fluid and iron overload) of blood transfusions. Hèbert et al. published their landmark study 15 years ago which demonstrated the lack of benefit of a liberal transfusion strategy over that of a restrictive one in critical care patients^5^. Since then, additional studies have been published demonstrating similar outcomes in other patient populations such as elderly patients undergoing orthopedic (e.g., hip replacement) surgery, pediatric patients, and patients with gastrointestinal bleeding^6-8^. Despite these data supportive of restrictive transfusion strategies along with the fact that published transfusion guidelines have been updated over the years to reflect a more conservative approach to blood utilization^9,10^, clinicians have been slow to adopt these transfusion practices and in many cases, transfusion practices remain inconsistent even amongst physicians of the same specialty practicing within a localized region or even within the same institution^11^. This review will further discuss issues surrounding blood utilization, including the safety and quality of the blood supply and patient blood management.

## 2. Safety of the blood supply

Over the years, the safety of the blood supply has dramatically improved. Early efforts to reduce risk focused on reliance on volunteer donors while screening out donors with high-risk behavior, such as male-to-male sex, for human immunodeficiency virus (HIV) and viral hepatitis (hepatitis B and hepatitis C) as well as the implementation of highly-sensitive testing to reduce the infectious window period, that is, the time between exposure to viral infection and ability of the blood test to detect the virus when the donor may be infectious though the test result is negative. Such strategies have reduced the risk of HIV and hepatitis C transmission through blood to about 1 in 2 million (or roughly the equivalent of the risk of getting struck by lightning)^12^. More recently, testing for West Nile virus and Chagas disease (Trypanosoma cruzi) has been added to address those emerging transmissible disease threats to the blood supply^13,14^, and standards were adopted for reducing the risk of bacterial contamination of platelet components (which must be stored at room temperature to maintain platelet hemostatic function)^15^. Still, there remain transmissible disease risks that are not tested for owing to the fact that U.S. Food and Drug Administration (FDA)-approved testing is unavailable. Perhaps most problematic in the U.S. is babesiosis, a parasitic agent that infects red blood cells à-la malaria but that is endemic in the northeastern U.S.; transmission of babesiosis through blood has been reported^16^, and the FDA may consider implementation of testing in the near future. Yet another transmissible agent for which testing is unavailable is variant Creutzfeldt-Jakob disease (vCJD, the prion agent of bovine spongiform encephalopathy or mad cow disease); human cases have been attributed to transfusion of blood components in the United Kingdom^17^. To address this, prospective blood donors in the U.S. who have traveled for a defined period of time to the U.K. (cumulative 3 month travel between 1980 and 1996) or western Europe (cumulative 5 years between 1980 and present) are currently excluded; however, this is problematic as a number of otherwise-eligible blood donors are unable to donate, representing a small but not insignificant loss of U.S. blood donors^18^. Transfusion-transmitted cases of hepatitis E virus have also been reported outside of the U.S., though testing for this agent is not currently performed either^19^.

As the risk of transmissible disease through blood transfusion has decreased, non-infectious risks have become more prominent. Transfusion-related acute lung injury (TRALI), defined as edema or fluid collection in the lungs during or shortly (within 6 hours) after transfusion not causally-linked to heart failure or other cause of lung injury unrelated to transfusion such as pneumonia, has been shown to be largely a result of antibodies to white blood cell antigens (known as human leukocyte antigen [HLA] antibodies) in female donors (because of exposure to HLA during pregnancy); as a result, the use of plasma components (which contain the HLA antibodies) from females has been restricted in the U.S. unless the female donor is known not to have ever been pregnant or is otherwise tested for the causative HLA antibodies since last pregnancy^20^. Transfusion of group ABO-incompatible blood is another non-infectious risk that has gained more recognition over time and is almost always due to human error such as error in collection of the crossmatch sample (i.e., the sample is collected from the wrong patient though labeled with the correct patient’s identification) or in patient identification at the time of blood transfusion (i.e., blood correctly crossmatched but given to wrong patient). Strategies to reduce this risk have been implemented by most hospital transfusion services, such as the requirement for a second sample to confirm the ABO blood type prior to crossmatch and strict blood administration policies for bedside patient identification at the time of transfusion. Nevertheless, TRALI and group ABO-incompatible transfusion occurrence estimates remain much greater than for transmission of HIV and hepatitis and are amongst the highest causes of transfusion-related fatalities^21,22^. Transfusion-associated graft-vs.-host disease (TA-GVHD) is yet another non-infectious complication of transfusion that is fatal^23^. However, though it is preventable through blood component irradiation which inactivates the causative immune cells (T4 lymphocytes), clinicians do not always order, or otherwise provide the patient’s condition to alert the hospital blood bank to prepare, irradiated blood for their at-risk patients (those who are immunocompromised or have certain conditions like leukemia or Hodgkin’s disease). Most hospital blood banks do not routinely maintain large inventories of irradiated blood since the irradiation process reduces the storage shelf life of the red blood cell products, nor do they have a blood irradiator device onsite which requires a significant amount space and carries a very high level of security concern to protect the radiation source^24^. Finally, in addition to all the above risks, blood transfusion can lead to the formation of unexpected alloantibodies to red blood cell antigens (i.e., non-ABO antibodies such as antibodies to the Rh blood group system) in recipients which can make future blood transfusions even more difficult in the way of finding compatible blood and result in hemolytic reactions (because of incompatibility related to the unexpected antibodies) as well as carrying the added risk in females of childbearing age of causing severe complications, even fetal death, during pregnancy (i.e., hemolytic disease of the fetus and newborn)^25^.

**Figure 1:**
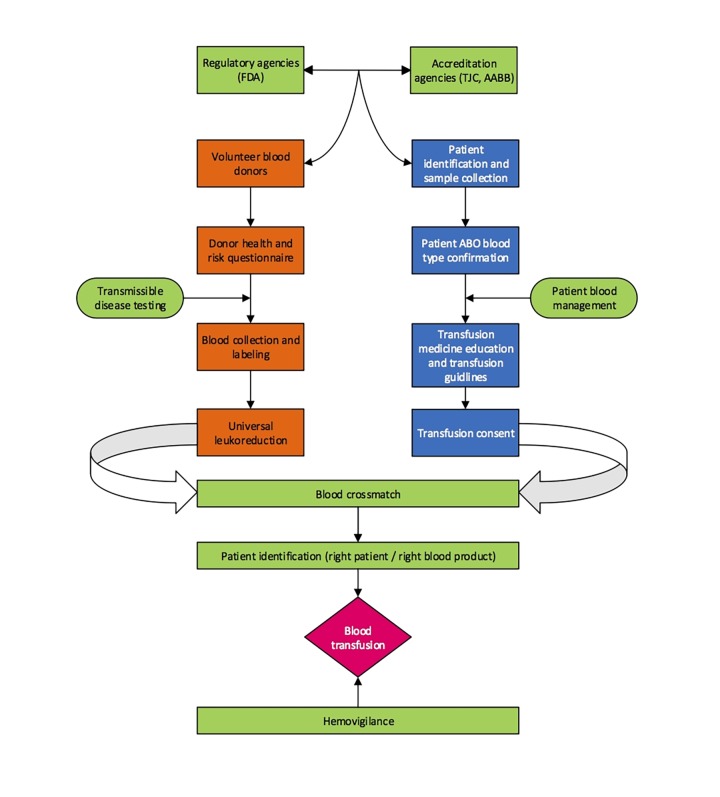
Transfusion safety schematic

In order to further improve blood safety across the nation, the U.S. Biovigilance Network, a collaboration between the U.S. Department of Health and Human Services, including the Centers for Disease Control and Prevention (CDC), and organizations that collect and transfuse blood, launched the National Hemovigilance Program in 2010^26^. The U.S. program followed that of many other developed countries, including France (where it was set into law by 1993) and the U.K.’s Serious Hazards of Transfusion (SHOT) program (which began in 1996)^27^. The International Hemovigilance Network defines hemovigilance as “surveillance procedures covering the whole transfusion chain, from collection of blood and its components to follow-up of recipients, intended to collect and assess information on unexpected or undesirable effects resulting from the therapeutic use of labile blood products and to prevent their occurrence or recurrence.”^27^ However, many hospital transfusion services across the U.S. have been slow to join the reporting system which remains voluntary, limiting its effectiveness. Transfusion services are required, however, to report serious reactions, especially those that result in fatality, to the FDA as well as to their local department of health in a number of states^28-30^. A transfusion safety schematic is presented in **Figure 1**.

## 3. Quality of the blood supply

Efforts have been put forth to improve the quality of the blood supply in other areas. One major improvement resulted when the system of labeling blood components converted from Codabar symbology to the ISBT 128 system which has the advantage of being a global system that is unique, comprehensive, and more accurate in its coding method^31^. A second major improvement has resulted from the trend toward universal leukoreduction, or depletion of white blood cells, from blood components. However, universal leukoreduction in the U.S. has lagged behind Europe and Canada which implemented leukoreduction much earlier partly in response to the risk of vCJD transmission (vCJD has been reported to be closely associated with the buffy-coat or white blood cell layer of blood though more recent evidence has shown infectivity in plasma and red blood cells)^32^. White blood cells, considered contaminants of red blood cell and platelet components, pose complications related to febrile transfusion reactions, alloimmunization (i.e., formation of HLA antibodies which can lead to transplant rejection), and transmission of some pathogens like cytomegalovirus (CMV, a common herpes virus that may cause complications like pneumonia in some at-risk patients such as those who are immunocompromised). Yet it is unclear what impact leukoreduction at the time of collection (i.e., pre-storage leukoreduction), which removes about 99% of the white blood cells and prevents buildup of white cell products (known as cytokines) during storage, will have on the mitigation of transfusion-related immunomodulation (TRIM). TRIM is the concept that blood transfusions weaken the immune system in recipients and increase the risk of postoperative infections and cancer recurrence^33^. Finally, there is some evidence that transfusion of older (greater than 14 days in storage) blood products compared with fresher ones (less than 14 days in storage) may also increase the risk of poorer hospital outcomes in some patient populations^34^.

## 4. Transfusion medicine education

Unfortunately, in light of the above risks and complications of blood transfusion, clinicians continue to receive little to no formal training in appropriate use of blood products and their practices continue to be principally based upon individual clinical experience^35^. In fact, assessment of overall transfusion medicine knowledge in our own hospital facilities showed poor baseline knowledge amongst physicians across different specialties and training levels^35^. As previously commented on in a published editorial, lack of transfusion medicine knowledge may possibly be the greatest obstacle toward making transfusion practices more consistent and in line with published guidelines and evidence-based medicine^36^.

## 5. Patient blood management

Patient blood management (PBM), as defined by the AABB, is “an evidence-based, multidisciplinary approach to optimizing the care of patients who might need transfusion. PBM encompasses all aspects of patient evaluation and clinical management surrounding the transfusion decision-making process, including the application of appropriate indications, as well as minimization of blood loss and optimization of patient red cell mass. PBM can reduce the need for allogeneic blood transfusions and reduce health-care costs, while ensuring that blood components are available for the patients who need them.”^37^ In addition to the AABB, the Joint Commission (TJC), a major organization that accredits healthcare organizations (formerly known as the Joint Commission on Accreditation of Healthcare Organizations or JCAHO), has drafted its own set of guidelines for PBM certification^38^. Previously, though, TJC published a set of blood management performance measures that hospital transfusion services could opt to use to improve their processes surrounding transfusion^39^. Measure PBM-02 RBC Transfusion Indication^39^ was in part developed around published data from our hospitals showing a significant correlation between lack of transfusion documentation in the patient medical record and failure to justify the transfusion as clinically necessary^40^. Transfusion services employ this measure to evaluate their blood utilization practices^41^, and we are currently planning a follow-up analysis in our own facilities. We further published data from our hospital facilities concerning Measure PBM-01 Transfusion Consent^39^demonstrating that information discussed with patients prior to transfusions tended to focus on the benefits of transfusion (namely, correction of anemia, which as noted above, may not actually be a benefit unless symptomatic) along with minor risks (such as febrile and allergic reactions) while omitting discussion of more significant risks such as hemolytic (incompatible blood) reactions, TRALI, volume overload, and transmission of HIV and hepatitis C^42^. PBM-01 is an important measure in that AABB standards require transfusion consent and in that the Institute of Medicine, in naming patient centeredness (defined as “providing care that is respectful of and responsive to individual patient preferences, needs, and values, and ensuring that patient values guide all clinical decisions”) as one of its 6 core attributes of a high-quality healthcare system, encourages dialogue between physicians and patients in the decision-making process^43^.

## 6. Future technology

Although PBM strategies should optimize and in many instances minimize the use of blood transfusions as such strategies gain prominence in hospitals throughout the U.S. over time, the need for blood will be ever present largely because of an aging population that is expanding and undergoing more medical procedures, including cardiothoracic surgery, joint replacement, transplant surgery, hemodialysis, and cancer treatment (chemo- and radiation therapy), which will require transfusion support in many cases. Yet the promise of blood substitutes, also known as “artificial blood”, which are really oxygen therapeutic agents rather than complete substitutes for blood, has proven to be quite elusive. Development of a therapeutic oxygen carrier, which ideally would be readily available, universally compatible, pathogen free, cost efficient with minimal side effects and a long shelf life, has taken two forms over the years: perfluorocarbon emulsions (PFC’s) and hemoglobin-based oxygen carriers (HBOC’s)^44,45^. PFC’s are chemically-inert, colorless, clear liquids that have the ability to dissolve large volumes of gases, including oxygen and carbon dioxide^44^. However, PFC’s are also water insoluble and must be emulsified for intravenous use, limiting their effectiveness due to low PFC content such that high concentrations of supplemental oxygen must be given in order to achieve a therapeutic effect. Fluosol-DA (Green Cross Corp., Osaka, Japan/Alpha Therapeutic, Los Angeles, CA), a first-generation PFC, received FDA approval in 1989 for use in coronary balloon angioplasty but was withdrawn from the market just 5 years later since it was found to be cumbersome to store (requiring frozen storage) and prepare for therapeutic use as well as the fact that improvements in angioplasty catheter technology eliminated the need for Fluosol^45,46^. Second generation PFC’s, which have higher PFC content, such as Oxygent (Alliance Pharmaceutical Corp., La Jolla, CA) and Oxyfluor (Hemagen, Inc., St. Louis, MO) were subsequently developed and tested but have not been approved by the FDA^45,46^. HBOC’s, on the other hand, are manufactured from human or bovine hemoglobin, and at least one recombinant (genetically-engineered) product was developed^45,47^. However, the main obstacle to the success of HBOC’s has been the fact that cell-free hemoglobin is quite toxic, causing increased vasoconstriction and vascular resistance leading to hypertension (due to scavenging of nitric oxide along the endothelial lining of the blood vessels where NO normally promotes vascular dilatation) and damage to the kidneys as well as gastrointestinal, hepatic, pancreatic, and neurological side effects^47^. In addition, HBOC’s have a rather short plasma half-life and have altered oxygen-binding properties^45^. Manufacturers have introduced various alterations to their proprietary HBOC’s in order to stabilize the cell-free hemoglobin and circumvent these problems including, intramolecular crosslinking, polymerization, pegylation, pyridoxilation, and encapsulation^45,46^. As such, an impressive array of HBOC’s have been in development, including PolyHeme (Northfield Laboratories, Evanston, IL), Hemolink (Hemosol, Mississauga, Ontario, Canada), HemAssist (Baxter Healthcare, Deerfield, IL), PHP (Apex Bioscience, Research Triangle Park, NC), Hemospan (Sangart, San Diego, CA), PEG-Hemoglobin (Enzon, Piscataway, NJ),

Hemopure (Biopure, Cambridge, MA), Oxyglobin (Biopure), and Optro (Somatogen, Boulder, CO), but none have achieved FDA approval, though Hemopure has been approved for use in South Africa since 2001^45^. In the end, it is likely that, if and when a so called blood substitute is approved by the FDA, the indications will be limited to extreme trauma or to situations in which blood transfusion may not be possible because of religious objection (as in Jehovah’s Witnesses who refuse transfusion with typical blood products) or because of difficulty in finding compatible blood. However, other applications may include use as a cardioplegic oxygen solution for open heart surgery, for treatment of ischemia in myocardial infarction and stroke patients, and as sensitizers to oxygenate solid tumors for increased response to chemo- or radiotherapy^45^. In other technology, artificial platelets (i.e., platelet substitutes) have been under development, but such development is still in the early stages^48^. Pathogen-inactivated cellular components, such as Cerus Corporation’s INTERCEPT Blood System (Cerus Corp., Concord, CA)^49^ have also not been fully developed as of yet while solvent-detergent-treated plasma has recently been reintroduced to the U.S. market, albeit by a different manufacturer (Octaplas, Octapharma AG, Lachen, Switzerland)^50^, after withdrawal of an earlier product, Plas+SD (VITEX, Watertown, MA) a decade ago after the FDA issued a Black Box warning contraindicating its use in liver transplant patients and those with severe liver disease (it must be noted here that Octaplas is manufactured using a different process and does not have such contraindication)^51^. Where technology perhaps has been most successful is in the development of factor concentrates. Both human plasma-derived and recombinant factor concentrates are available for use today to treat hemophilia and other coagulation disorders (see **Table 1**). Unfortunately, because human plasma-derived factor concentrates are manufactured from pooled plasma from thousands of donors, many of the early (1970’s-1980’s) hemophilia factor concentrates were tainted with HIV and hepatitis leading to the deaths of many hemophiliacs. By the mid 1980’s, however, heat treatment and later other viral-inactivation steps such as solvent-detergent treatment were incorporated into the manufacturing processes of the plasma-derived concentrates along with improved donor screening and transmissible disease testing^52^. Recombinant factor VIII and factor IX products became available in the 1990’s; since then, other recombinant factors have been approved (**Table 1**). These concentrates, though, are much more costly than conventional blood products (costing on average several thousand dollars per dose vs. several hundred dollars for a typical dose [several units] of transfused plasma). Yet more problematic is the clinical use of these products for the treatment of conditions for which there is little to no evidence of benefit and for which they were not intended for use by the FDA (i.e., off-label use). As an example, recombinant factor VIIa (NovoSeven RT, Novo Nordisk, Bagsvaerd, Denmark), intended for use in certain patients with hemophilia^53^, was used for treatment of intracerebral bleeding after one study published in the New England Journal of Medicine showed promising data^54^. Enthusiasm for such use diminished, however, after a follow-up study failed to demonstrate a survival benefit even though the study did demonstrate effectiveness of the product in controlling the bleeding into the brain^55^. Nevertheless, off-label use for recombinant factor VIIa and other concentrates persists with significant costs, uncertain benefits, and possible thomboembolic risks due to the high-clotting potency of these products.

**Table 1:** Factor Concentrates Licensed in the United States* *As of March 2014; list may not be all-inclusive; consult manufacturer package inserts for further details on products. GTC = GTC Biotherapeutics, Inc., Framingham, MA Grifols = Grifols Biologicals, Inc., Los Angeles, CA Baxter = Baxter Healthcare Corp., Westlake Village, CA CSL Behring = CSL Behring GmbH, Marburg, Germany Novo Nordisk = Novo Nordisk A/S, Bagsvaerd, Denmark Octapharma = Octapharma , Lachen, Switzerland Bayer = Bayer Healthcare, Tarrytown, NY Pfizer = Pfizer Inc., Philadelphia, PA

Factor	Human Plasma / Recombinant	Product Name	Manufacturer	Indications
Antithrombin III	Recombinant	ATryn	GTC	Hereditary antithrombin III deficiency
	Human Plasma	Thrombate	Grifols	Hereditary antithrombin III deficiency
Protein C	Human Plasma	Ceprotin	Baxter	Severe congenital Protein C deficiency
Fibrinogen	Human Plasma	RiaSTAP	CSL Behrig	Congenital fibrinogen deficiency
Factor VII (activated)	Recombinant	NovoSeven RT	Novo Nordisk	Hemophilia A or B with inhibitors; acquired hemophilia; congenital Factor VII deficiency
Factor VIII / von Willebrand factor complex	Human Plasma	Alphanate; Humante-P	Grifols; CLS Behring; respectively	Hemophilia A; von Willebrand disease
		Wilate	Octapharma	von Willebrand disease
Factor VIII	Recombinant	ADVATE; Helixate FS; Kogenate FS; Recombinate; Xyntha	Baxter; Bayer; Bayer; Baxter; Pfizer respectively	Hemophilia A
	Human Plasma	HEMOFIL M; Koate-DVI; Monoclate-P	Baxter; Grifols; CLS Behring respectively	Hemophilia A
Factor IX	Recombinant	BeneFIX; Rixubis	Pfizer; Baxter respectively	Hemophilia B
	Human Plasma	AlphaNine SD; Mononine	Grifols; CLS Behring respectively	Hemophilia B
Factor VIII inhibitor bypassing activity (Factors II, IX, X, VIIa complex)	Human Plasma	FEIBA VH	Baxter	Hemophilia A or B with inhibitors
Factor IX complex (Factors II, IX, X, low Factor VII)	Human Plasma	BEBULIN; Profilnine SD	Baxter; Grifols respectively	Hemophilia B
Prothrombin complex (Factors II, VII, IX, X, Protein C, Protein S, Antithrombin III)	Human Plasma	Kcentra	CLS Behring	Bleeding due to warfarin anticoagulation
Factor XIII	Recombinant	Tretten	Novo Nordisk	Congenital Factor XIII deficiency
	Human Plasma	Corifact	CSL Behring	Congenital Factor XIII deficiency

## 7. Concluding remarks

Transfusion medicine continues to evolve with improvements in the quality and safety of blood components to minimize transfusion risks. Educating clinicians to follow appropriate transfusion practices under established blood management protocols is also of high importance, particularly since the accrediting agencies (AABB and TJC) will hold hospitals more accountable for their transfusion practices and in the light that newer, more potent, and more costly blood products, such the factor concentrates noted above, are becoming much more available and in demand. The search for a blood substitute continues but will likely have only limited applications when finally approved. Though many advances have been made in the field of transfusion medicine to date, a number of questions remain incompletely answered, such as the benefits vs. risks of more restrictive transfusion practices in higher-risk patients (i.e., those with underlying cardiovascular disease), the risks of transfusing older vs. fresher blood, the long term risks of blood transfusion on the immune system (TRIM), and the best practices to prevent established risks such as TRALI as well as questions about emerging risks. Further studies will be necessary and in some cases are ongoing to provide insight into these and other issues surrounding the transfusion of blood products. Ultimately though, we could not agree more with Menitove et al.^56^ who advocate for a risk-based decision-making approach toward blood safety, noting that “some blood product safety initiatives cost more than 10 times the currently accepted threshold of up to $100,000/quality-adjusted life year gained for other medical interventions” in the drive to attain a zero-risk blood supply.

- **Although blood transfusions are commonly used to treat anemia, restrictive transfusion practices (i.e., fewer transfusions) have been shown to be as effective if not better than liberal transfusion practices.**
- **Improved donor screening and transmissible disease testing, particularly for HIV and hepatitis C, vastly increased the safety of the blood supply**
- **More recent efforts to increase safety have largely focused on noninfectious risks and bacterial contamination of blood products.**
- **PBM strategies such as: preoperative anemia management, use of surgical techniques to minimize blood loss, or recover shed blood (intraoperative cell salvage), can minimize the need for blood transfusions.**
- **Blood substitutes, really oxygen therapeutic agents, though in development for many years are not approved for use in the U.S. and cannot replace the need for blood.**
- **Should all possible blood safety initiatives, no matter how remote the risk, be implemented at any cost? **
